# Genetic complexity underlies clinical heterogeneity: YWTD β-propeller mutations and second-hit modifier mutations in LRP6-related tooth agenesis and ectodermal dysplasia in human

**DOI:** 10.1016/j.gendis.2025.101541

**Published:** 2025-01-22

**Authors:** Xinxin Dong, Miao Yu, Zhaoyang Jia, Jinye Bai, Jing Zhang, Shengnan Ling, Limin Guan, Yi Lu, Dong Han, Xiwen Gu

**Affiliations:** a Key Laboratory of Shaanxi Province for Craniofacial Precision Medicine Research, Research Center of Stomatology, College of Stomatology, Xi'an Jiaotong University, Xi'an, Shaanxi 710004, China; b Department of Prosthodontics, College of Stomatology, Xi'an Jiaotong University, Xi'an, Shaanxi 710004, China; c Department of Prosthodontics, Peking University School and Hospital of Stomatology & National Center for Stomatology & National Clinical Research Center for Oral Diseases & National Engineering Research Center of Oral Biomaterials and Digital Medical Devices & Central Laboratory, Peking University School and Hospital of Stomatology, Beijing 100081, China; d Department of Stomatology, Xi'an Children's Hospital, Xi'an, Shaanxi 710003, China; e State Key Laboratory of Holistic Integrative Management of Gastrointestinal Cancers and Department of Pathology, Xijing Hospital and School of Basic Medicine, Fourth Military Medical University, Xi'an, Shaanxi 710000, China

Hereditary disorders often present remarkable genetic and clinical heterogeneities, but bridging the gap between pathogenic mutation and clinical consequences remains challenging. One plausible explanation is the presence of second-hit modifier mutations, which could create additional genetic complexity to contribute to clinical heterogeneities. We propose to trace modifier mutations by focusing on single-pedigree patients who present divergent clinical phenotypes; this scenario would ensure shared pathogenic mutations and highly related genetic makeups, thus simplifying the detection of second-hit modifiers. An appropriate window to test this idea lies in the case of low-density lipoprotein receptor-related protein 6 (LRP6), which encodes one essential co-receptor of Wnt signaling and is involved in multiple human diseases. In particular, LRP6 has been discovered as a frequent pathogenic gene for tooth agenesis (absence of one or more permanent teeth) or in rare cases for ectodermal dysplasia where affected members showed variable abnormalities of teeth, hair, and sweat glands.^1^ Yet the pathogenic mechanism of LRP6-related tooth agenesis and the genetic cause for its clinical heterogeneity remain unclear. Here, we report a novel LRP6 C1032F mutation in one ectodermal dysplasia family, highlighting YWTD-motif targeting as a generalized mechanism in LRP6-associated tooth agenesis and involvement of second-hit modifier mutations in LRP6-associated clinical heterogeneity.

We first examined one ectodermal dysplasia family with autosomal dominant transmission. In this family, a 13-year-old male proband (II:1) presented severe tooth agenesis with missing 13 permanent teeth (third molars excluded) and retention of multiple deciduous teeth (Fig. 1A, B). The proband also complained of several additional symptoms consistent with ectodermal dysplasia, including heat intolerance, dry eye, and dry mouth (Table S1). In addition, the proband presented distinctive facial features, including a concaved facial profile indicative of skeletal class III malocclusion, a moderately depressed nasal bridge, and a loose and everted upper lip with skin discoloration (Fig. 1C). The proband's mother (I:2) presented missing of 8 permanent teeth and similarly concaved facial profile, but differed significantly from the proband due to obvious frontal bossing and lack of heat intolerance, hinting potential involvement of modifier mutations (Table S1).

**Figure 1:**
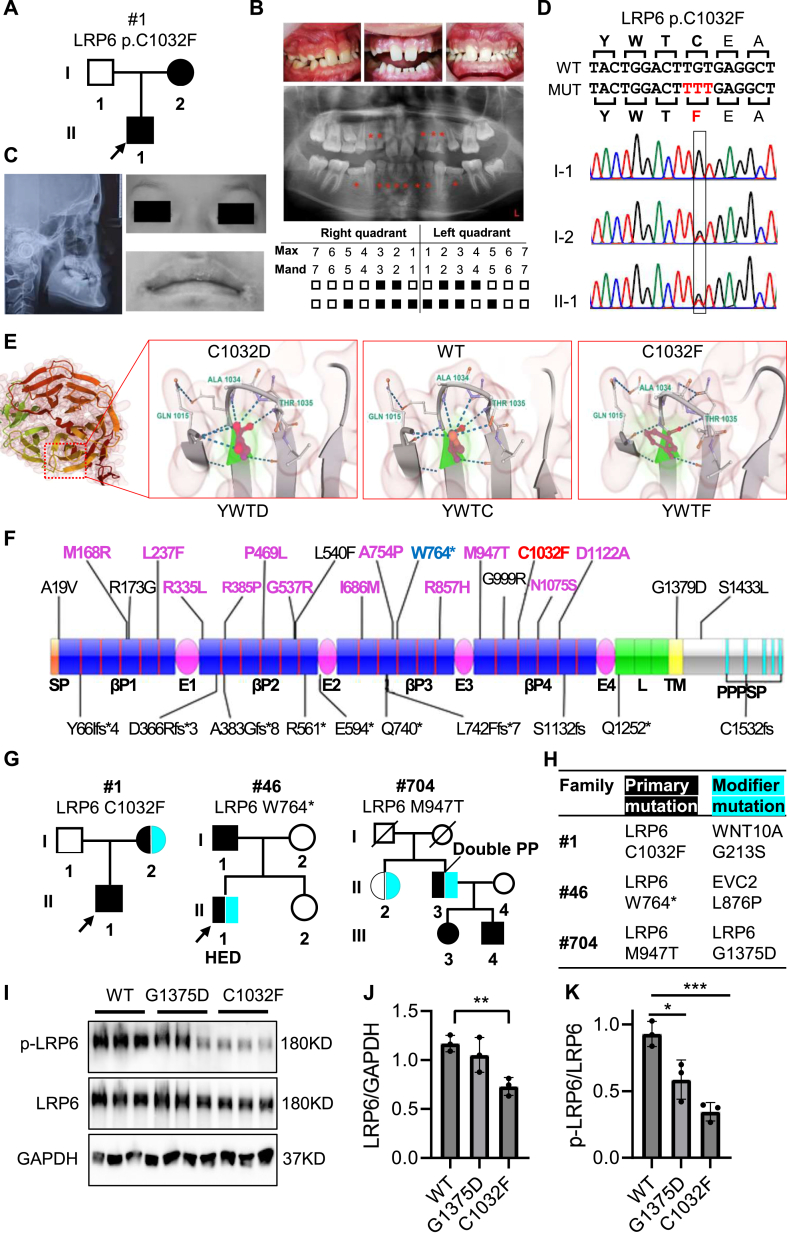
YWTD β-propeller mutations and second-hit modifier mutations in LRP6-related tooth agenesis and ectodermal dysplasia. **(A)** Pedigree of family #1 with ectodermal dysplasia. The black circle/square represents patients and the arrow indicates the proband. **(B)** Intraoral tooth photographs (top panel), panoramic radiograph (middle panel), and tooth schematic diagram (bottom panel) of family #1 proband (II-1) with 13 congenital permanent tooth agenesis (excluding third molars). The asterisks and solid squares indicate missing permanent teeth. Max, maxillary; Mand, mandibular. **(C)** The lateral cephalometric radiograph of the proband (II-1) shows an overall concaved facial profile with midface hypoplasia and protruding chin. The proband also had a moderately depressed nasal bridge and a loose and everted upper lip with skin discoloration at the oral commissure. **(D)** Sanger sequencing showed a heterozygous *LRP6* missense mutation (c.3095G > T; p.C1032F) in the proband (II-1) and proband's mother (I-2). **(E)***In silico* structural modeling of the wild-type (YWTC, C1032), prototype (YWTD, C1032D), and mutant LRP6 variants (YWTF, C1032F) at the C1032 site. The dashed lines indicate hydrogen bonding. Three residues (Gln 1015, Ala 1034, and Thr 1035) forming hydrogen bonds with the side chain of C1032 are highlighted. The overall six-bladed structure of LRP6 fourth beta-propeller is shown. **(F)** The diagram of LRP6 protein structure with 31 known tooth-agenesis-related mutations showing frequent targeting of YWTD motif by LRP6 missense mutations. Each YWTD motif is represented as a red vertical line. Missense mutations that interact with nearby YWTD motifs are labeled in purple, and frame-shift (fs) and stop-gain (∗) mutations are labeled at the bottom. SP, signal peptide; βP1–βP4, four beta-propeller domains; E1–E4, four EGF-like domains; L, LDL-type repeat; TM, transmembrane domain; PPPSP, intracellular PPPSP motifs. **(G**, **H)** Identification of potential modifier mutations in three LRP6 families (#1, #46, and #704) with intra-family clinical heterogeneities. The pedigree diagram (G) shows different intra-family patients harboring genetic heterogeneity, depicted as the shared LRP6 primary mutation (black-filled) but unshared modifier mutations (cyan-filled). A simplified summary of primary and modifier mutations in three families is shown in (H). HED, hypohidrotic ectodermal dysplasia; Double PP, preaxial polydactyly affecting both hands. **(I–K)***In vitro* validation of impaired Wnt signaling in LRP6 C1032F pathogenic and G1375D modifier mutant constructs. 293T cells were transiently transfected with LRP6 vectors expressing wild-type (lane 1–3), G1375D (lane 4–6), or C1032F variant (lane 7–9). Representative Western blot (I) and quantification (J, K) showed that the LRP6 C1032F pathogenic mutation exhibited reduced LRP6 protein abundance as well as significantly diminished LRP6 phosphorylation at the conserved cytoplasmic PPPSP motifs, which serves as a reliable marker for impaired Wnt signaling, whereas the LRP6 G1375D mutation exhibited only moderate reduction of LRP6 phosphorylation, supporting its potential modifier role. GAPDH served as a loading control. The experiment was repeated twice with similar results. The data are represented as mean ± standard deviation. Statistical significance was determined using an unpaired student's *t*-test. ∗*p* < 0.05, ∗∗*p* < 0.01, ∗∗∗*p* < 0.001.

Whole exome sequencing and Sanger sequencing identified a novel heterozygous missense mutation in exon 14 of *LRP6* (c.G3095T; p.C1032F) in both the proband (II:1) and his mother (I:2) (Fig. 1D). Notably, this LRP6 C1032F mutation targeted a unique YWTC motif (Tyr-Trp-Thr-Cys abbreviation), the variant form of classical β-propeller YWTD (Tyr-Trp-Thr-Asp) motif. This motif constitutes the most salient feature of the LRP6 extracellular structure because the LRP6 extracellular region contains four β-propeller modules (βP1–βP4) with each harboring six YWTD repeats, reaching 24 YWTD repeats in total (Fig. 1E, F).^2^ To explore its functional impact, we performed *in silico* structural modeling of the wild-type (C1032), the mutant (C1032F), and the prototypic variant (C1032D), using the crystal structure of LRP6-PE3PE4 (PDB A40P) as a template. As shown in Figure 1E, the wild-type C1032 residue sits at the top of the second β-sheet and forms extensive hydrogen bonds with multiple neighboring residues (Q1015, A1034, T1035, N1036, and V1037). Likewise, the prototypic C1032D variant was also capable of forming highly identical hydrogen bonds. However, the C1032F mutant, which substituted cysteine for aromatic and hydrophobic phenylalanine, was very inert in hydrogen bonding, suggesting a deleterious and destabilizing effect. Further analysis revealed that the C1032F-propeller blade has high evolutionary conservation, especially at the YWTD motif and its surrounding residues that directly form hydrogen bonding with the wild-type C1032 site (Fig. S1A). These findings are consistent with the central role of the YWTD motif in organizing and stabilizing the β-propeller structure. Moreover, differential motif analysis revealed that different β-propeller modules in LRP5/6 differed in YWTD motif conservation, with the fourth β-propeller showing the greatest YWTD motif variability (Fig. S1B). To validate its deleterious effect, we established the LRP6 wild-type and C1032F construct for *in vitro* functional assay. Accordingly, the C1032F mutant construct showed reduced LRP6 protein abundance and greatly reduced LRP6 protein phosphorylation (compared with the wild-type construct), consistent with a deleterious and destabilizing effect (Fig. 1I–K). These findings established LRP6 C1032F mutation as a novel and dominant pathogenic mutation for tooth agenesis.

To further explore the role of YWTD targeting in LRP6-associated tooth agenesis, we collected 31 reported LRP6 tooth-agenesis mutations and examined their potential YWTD association using LRP6 crystal structures as reference (LRP6-PE1PE2, 3S94; LRP6-PE3PE4, 4A0P). Around one-third of these mutations were frame-shift or stop-gain mutations, which were excluded from structural analysis because of their general loss-of-function nature. Notably, most of the tooth agenesis-related LRP6 missense mutations were distributed within the β-propeller modules, whereas the four subsequent EGF-like domains (E1–E4) remained untargeted. Moreover, three missense mutations (R335L, C1032F, and N1075S) were located exactly at the YWTD motifs, while most of the remaining missense mutations were located at positions that form close coupling to YWTD motifs in structure (Fig. 1F and Table S2). These findings support that YWTD targeting and β-propeller destabilization may represent a generalized mechanism in LRP6-related tooth agenesis.

To explore whether second-hit modifier mutations exist to account for clinical heterogeneities between the proband (II-1) and his mother (I-2) in family #1, we searched for patient-private mutations using stringent bioinformatic criteria including rareness, evolutionary conservation, validated or at least predicted damaging effect, and occurrence in genes causative for relevant diseases. Interestingly, we identified in the mother (I-2) only, a deleterious variant targeting a conserved glycine residue in Wnt ligand gene WNT10A (Wnt family member 10A) (c.637G > A, p.G213S; Fig. S2A). Because the WNT10A G213S mutation has been reported as one of the compound pathogenic WNT10A mutations for tooth agenesis (pathogenic when combined with a second WNT10A mutation) and has been demonstrated to impair Wnt signaling in zebrafish-based functional assay,^3^ these findings support WNT10A G213S as a second-hit modifier mutation in family #1 (Fig. 1G, H).

We have previously reported two additional LRP6 tooth agenesis families (#46 and #704) with remarkable intra-family clinical heterogeneities (Fig. 1G, H).^4^^,^^5^ To further explore the involvement of modifier mutations, we reanalyzed the whole exome sequencing data for patient-private mutations using the above-mentioned strategy. In family #46, the proband (II-1) exhibited hypohidrotic ectodermal dysplasia but the father (I-1) presented only mild tooth agenesis; and we identified in the proband only, a private heterozygous mutation targeting EVC2 (c.T2627C, p.L876P, leucine-to-proline) (Fig. 1G, H; Fig. S2B), which encodes a positive modulator of the Hedgehog signaling. EVC2 mutations are involved in tooth agenesis and ectodermal dysplasia.^1^ In family #704, the proband's grandfather (II-3) exhibited preaxial polydactyly of both hands (double PP) but the proband's mother (III-3) had left-hand polydactyly only; and we identified in the grandfather (II-3) only, a private LRP6 heterozygous mutation (c.G4124A, p.G1375D, glycine-to-aspartate) targeting a conserved GxxxG motif within LRP6 transmembrane domain (Fig. 1G, H; Fig. S2C). By *in vitro* functional assay, we found that the G1375D mutant construct had normal LRP6 protein abundance but a substantial reduction of LRP6 protein phosphorylation (Fig. 1I–K), which is consistent with the proposed role for transmembrane GxxxG motif in mediating helix–helix association (self-dimerization). Notably, the *in vitro* assay also supports a weaker damaging effect for G1375D mutation than C1032F mutation (Fig. 1I–K). In addition, the LRP6 G1375D mutation was not self-pathogenic because it was also observed in one healthy sibling (II-2) (Fig. 1G, H). The weaker damaging effect and the occurrence in healthy carriers support LRP6 G1375D mutation as potential second-hit modifier mutations. Overall, our findings provide novel insights into LRP6-associated tooth agenesis and support that the co-occurrence of LRP-related tooth agenesis with additional rare phenotypes may be caused by second-hit rare modifier mutations, providing a paradigm of genetic heterogeneity underlying clinical heterogeneity in familial genetic disorders.

## Ethics declaration

The written informed consent from all the participants for the use of blood or saliva samples, clinical data, and publication of their photographs were obtained in accordance with the Declaration of Helsinki. All experiments were approved by the Ethics Committee of Xi'an Jiaotong University College of Stomatology (2020NO.012).

## Funding

This study was supported by the 10.13039/501100001809National Natural Science Foundation of China (No. 32070690 to X.G.) and the 10.13039/501100007128Natural Science Foundation of Shaanxi Province, China (No. 2020JM-412 to X.G.).

## CRediT authorship contribution statement

**Xinxin Dong:** Data curation, Formal analysis, Writing – original draft. **Miao Yu:** Data curation, Formal analysis, Resources, Writing – review & editing. **Zhaoyang Jia:** Data curation, Formal analysis, Investigation, Software, Writing – review & editing. **Jinye Bai:** Formal analysis, Validation, Writing – review & editing. **Jing Zhang:** Formal analysis, Writing – review & editing. **Shengnan Ling:** Formal analysis, Methodology, Writing – review & editing. **Limin Guan:** Formal analysis, Investigation. **Yi Lu:** Conceptualization, Investigation, Writing – review & editing. **Dong Han:** Formal analysis, Investigation, Resources, Supervision, Writing – review & editing. **Xiwen Gu:** Conceptualization, Formal analysis, Supervision, Visualization, Writing – review & editing.

## Conflict of interests

The authors declared no conflict of interests in this study.
