# Supplementary material for: Genetic complexity underlies clinical heterogeneity: YWTD β-propeller mutations and second-hit modifier mutations in LRP6-related tooth agenesis and ectodermal dysplasia in human
Source: Genes Dis. 2025 Jan 22;12(6):101541. doi: 10.1016/j.gendis.2025.101541 (PMC12275974; doi:10.1016/j.gendis.2025.101541)
Supplement: Multimedia component 1 [file mmc1.docx]

**Rapid communication supplemental data**

**Title:** Genetic complexity underlies clinical heterogeneity: YWTD β-propeller mutations and second-hit modifier mutations in LRP6-related tooth agenesis and ectodermal dysplasia in human

**Table S1.** Differential clinical features between the mother (I-2) and son (II-1) in family #1 as determined by clinical examination and medical questionnaire.

| **Clinical features** | **Description** | **Mother(I-2)** | **Son (II-1)** |
| --- | --- | --- | --- |
| Dental | Missing of permanent teeth | ✓ | ✓ |
|  | Retention of primary teeth | ✓ | ✓ |
| Skin | Dry skin with periorbital hyperpigmentation | ✓ | ✗ |
|  | Recurrent childhood skin lesions | n/a | ✓ |
| Hair | Sparse hair | ✗ | ✗ |
|  | Sparse eyebrow | ✗ | ✗ |
| Facial | concaved facial profile | ✓ | ✓ |
|  | Moderate frontal bossing | ✓ | ✗ |
|  | Depressed nasal bridge | ✓ | ✓ |
| Sweat | Heat intolerance | ✗ | ✓ |
|  | Decreased sweating | ✗ | n/a |
| Nail | Nail anormaly | ✗ | ✗ |
| Other exocrine   glands | Dry mouth | ✗ | ✓ |
|  | Dry eyes | ✗ | ✓ |
|  | Chronic childhood nasal drainage/blockage | n/a | ✓ |

**Table S2.** Summary of YWTD motif involvement for 19 LRP6 missense mutations associated with tooth agenesis.

| **Gene** | **Nucleotide change** | **Protein change** | **Phenotype** | **Location** | **YWTD-association** | **PMID reference** |
| --- | --- | --- | --- | --- | --- | --- |
| LRP6 | c.56C>T | A19V | Tooth agenesis, selective, 7 | N-terminal signal peptide | No | 26387593 |
| LRP6 | c.503T>G | M168R | Familial tooth agenesis | β-propeller 1 (βP1) | Interacting with the third YWTD of βP1 via hydrogen bond | 34834569 |
| LRP6 | c.517C>G | R173G | Tooth agenesis | β-propeller 1 (βP1) | Interacting with the fourth YWTD of βP1 via indirect hydrogen bond | 26963285 |
| LRP6 | c.711G>T | L237F | Non-syndromic tooth agenesis | β-propeller 1 (βP1) | Immediate neighbor of the sixth YWTD of βP1 | 32844563 |
| LRP6 | c.1004G>T | R335L | Orofacial cleft | β-propeller 2 (βP2) | Targeting the first atypical YWTD of βP2 | 26963285 |
| LRP6 | c.1154G>C | R385P | Tooth agenesis, selective, 7 | β-propeller 2 (βP2) | Interacting with the second YWTD of βP2 via backbone hydrogen bond | 34759310 |
| LRP6 | c.1406C>T | P469L | Tooth agenesis, selective, 7 | β-propeller 2 (βP2) | Spatial proximity to the fourth YWTD of βP2 | 26963285 |
| LRP6 | c.1609G>A | G537R | Tooth agenesis | β-propeller 2 (βP2) | Interacting with the sixth YWTD of βP2 via backbone hydrogen bond | 26963285 |
| LRP6 | c.1620G>T | L540F | Orofacial cleft | β-propeller 2 (βP2) | Not found | 26963285 |
| LRP6 | c.2058C>G | I686M | Orofacial cleft | β-propeller 3 (βP3) | Interacting with the third YWTD of βP3 via hydrophobic interaction | 26963285 |

**Table S2.** Continued.

| **Gene** | **Nucleotide change** | **Protein change** | **Phenotype** | **Location** | **YWTD-association** | **PMID reference** |
| --- | --- | --- | --- | --- | --- | --- |
| LRP6 | c.2260G>C | A754P | Familial tooth agenesis | β-propeller 3 (βP3) | Interacting with the fourth YWTD of βP3 via hydrogen bond | 34834569 |
| LRP6 | c.2570G>A | R857H | Tooth agenesis | β-propeller 3 (βP3) | Next to the sixth YWTD of βP3 | 34306029 |
| LRP6 | c.2840T>C | M947T | Tooth agenesis, selective, 7 | β-propeller 4 (βP4) | Interacting with the first atypical YWTD of βP4 via backbone hydrogen bond | 34759310 |
| LRP6 | c.2995G>C | G999R | Orofacial cleft | β-propeller 4 (βP4) | Not found | 26963285 |
| LRP6 | c.3095G>T | C1032F | Tooth agenesis, ectodermal dysplasia | β-propeller 4 (βP4) | Targeting the third YWTD motif of βP4 | This paper |
| LRP6 | c.3224A>G | N1075S | Familial tooth agenesis | β-propeller 4 (βP4) | Targeting the fourth YWTD motif of βP4 | 34834569 |
| LRP6 | c.3241C>G | D1122A | Orofacial cleft | β-propeller 4 (βP4) | Interacting with the fifth YWTD of βP4 via hydrogen bond | 26963285 |
| LRP6 | c.4136G>A | G1379D | Orofacial cleft, Tooth agenesis | Transmembrane domain | No | 26963285 |
| LRP6 | c.4298C>T | S1433L | Orofacial cleft, Tooth agenesis | Intracellular domain | No | 26963285 |


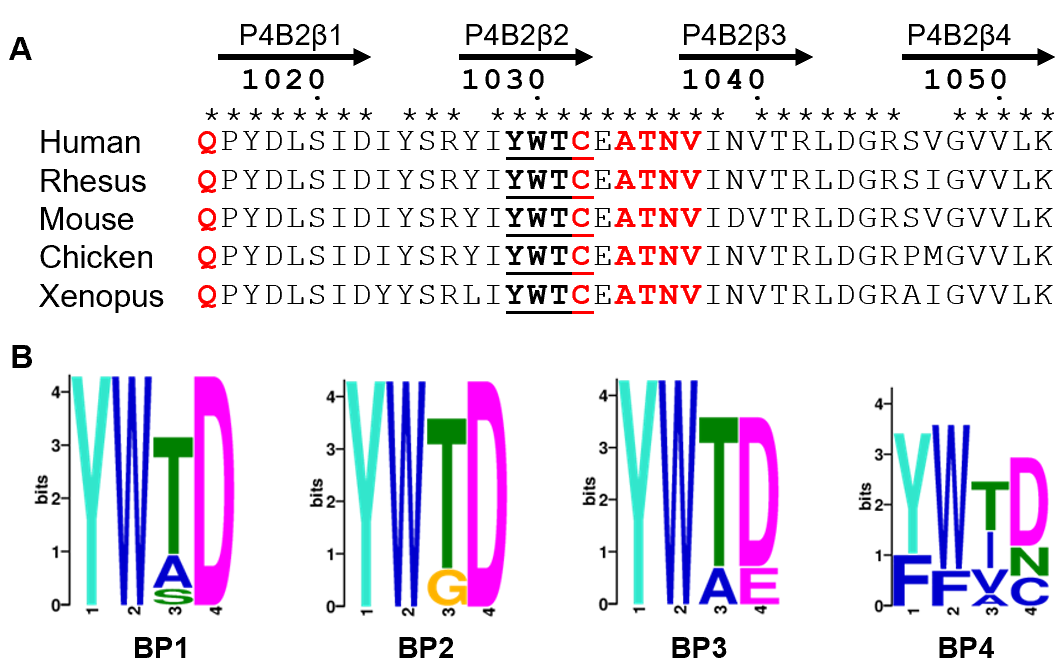
**Fig.S1 Conservation and motif analysis of LRP6 C1032F mutation.**

**(A**) Multiple sequence alignment of human LRP6 and their homologs around the C1032 mutation site, showing high evolutionary conservation especially around the YWTC motif (underlined). In P4B2β1-4, P4 represents the fourth β-propeller (BP4), B2 represents the second blade, and β1-4 represents the four beta-sheets constituting the blade. Surrounding residues that form hydrogen bonds with the C1032 site are labeled in red.

(**B**) Comparative analysis of YWTD motif variability across the four beta-propeller domains (BP1-BP4) of human and mouse LRP5/6 proteins. The motif variability was visualized using the MEME suite tool[1].


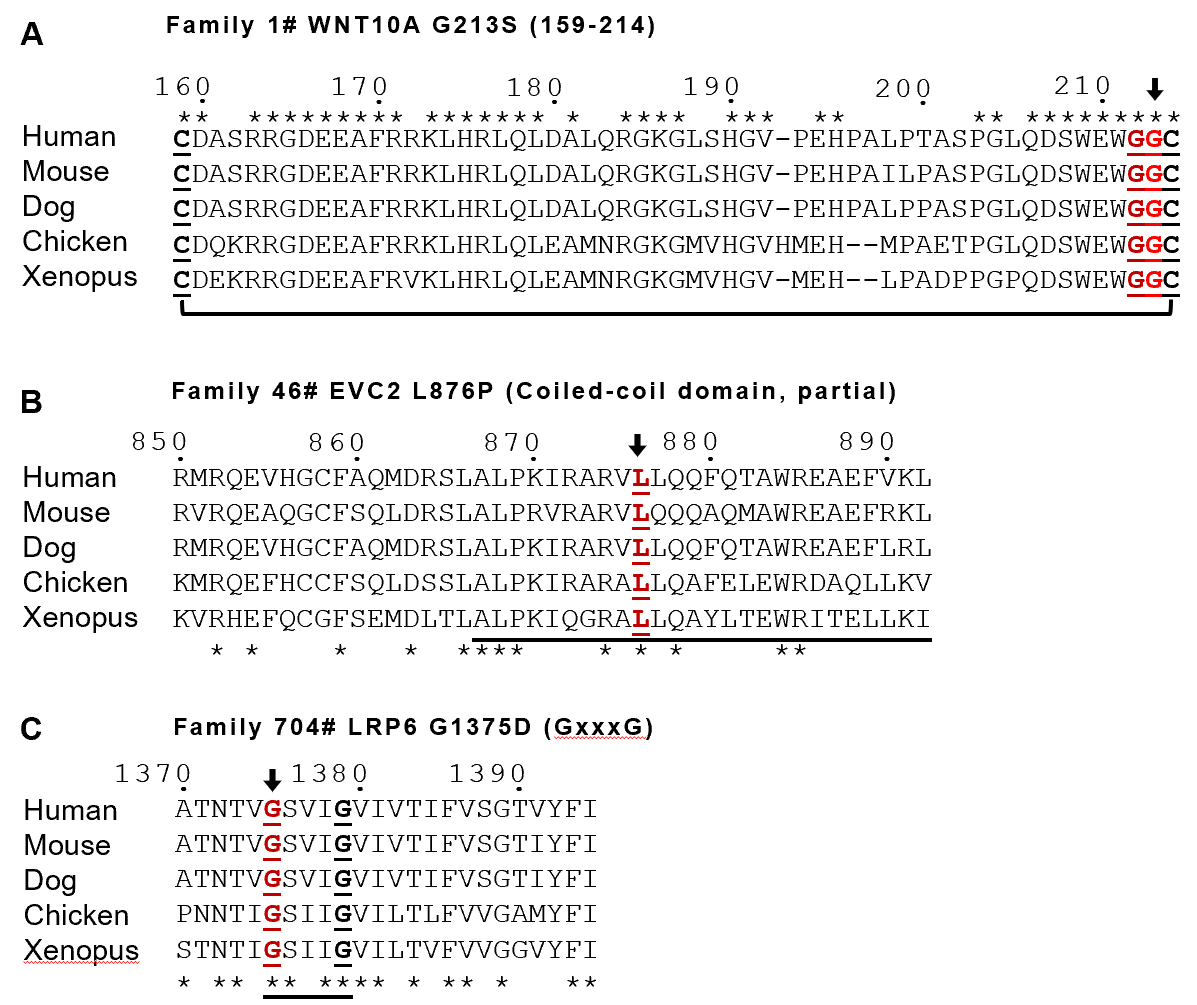
**Fig.S2 Structure-guided conservation analysis of the potential modifier mutations in three families.** Asterisks indicates conserved redidues.

**(A)** Alignment of human WNT10A protein surrounding the G213S mutation (residue 159-214; NP_079492.2) in family 1#. The AlphaFold model[2] of WNT10A structure (Accession: Q9GZT5) supports a disulfide-bond linkage between two separated cysteine residues (C159, C214; labeled with bottom black link). The G213S mutation (black arrow) targets one of the two consecutive glycine residues that immediately precedes the C214 residue. Presumably, the G213S mutation could impair the disulfide stability between C159-C214 residues due to steric hindrance.

**(B)** Alignment of human EVC2 protein surrounding the L876P mutation (residue 850-892; NP_667338.3) in family 46#. AlphaFold model of EVC2 structure (Accession: Q86UK5) supports a stable α-helix for the 867-892 fragment (labeled with bottom black line) with high confidence (pLDDT score > 90). The L876P mutation (leucine-to-proline; black arrow) would in theory compromise this local α-helix due to the rigid ring structure of proline residue.

**(C)** Alignment of the transmembrane domain (TM) of human LRP6 protein surrounding the G1375D mutation (residue 1370-1394; NP_002327) in family 704#. The highly conserved G1375-G1379 sites constitute the GxxxG motif that is involved in LRP6 homo-dimerization and subsequent signal transduction [3, 4].

**Detailed Materials and Methods**

**Patients**

One family with severe missing of permanent teeth were recruited from the Department of Prosthodontics at Xi’an Jiaotong University Hospital of Stomatology. The affected family members presented several clinical features reminiscent of ectodermal dysplasia, including oligodontia, self-reported dry eye and dry mouth, and concaved facial profile etc. They also filled out a self-made questionnaire to record clinical phenotypes and potential family histories. Written informed consent for participation and sample collection was obtained from all participants and/or their legal guardians in accordance with the Declaration of Helsinki. Detailed intraoral and radiographic examinations were performed by a prosthodontist to determine the status of dentition and missing permanent teeth.

Two additional families, one with hypohidrotic ectodermal dysplasia and the other with tooth agenesis plus polydactyly have been reported previously [5, 6], and were reanalyzed to explore potential modifier mutations underlying their intra-family clinical variabilities. This study was approved by the Ethical Committee of the Hospital of Stomatology Xi’an Jiaotong University (2020NO.012).

**Whole exome sequencing**

Genomic DNA of the proband and their parents were extracted from peripheral blood lymphocytes using the TIANamp Blood DNA Kit (Tiangen, China) and sent for whole exome sequencing (Anoroad, China). Briefly, sequencing libraries were prepared using the Agilent SureSelect XT Reagent kit and captured using the SureSelect Human All Exon V6 kit. The quality of DNA library was assessed using Qubit Fluorometer 3.0 and Agilent 2100 bioanalyzer respectively. Paired-end sequencing was conducted using the NovaSeq S2 platform (Illumina) with a read length of 150bp. With a sequencing yield of ~10 Gb per sample, the average target sequencing depth was ~100x per sample, with 97.7% of the targeted bases reached a depth of 20x and over 93.5% of clean reads reaching Q30 quality score.

**Variant prioritization and discovery**

A comprehensive prioritization pipeline was used to identify candidate pathogenic and modifier mutations. We first excluded silent variants, intronic variants, and common missense variants with a minor allele frequency (MAF) ≥0.05 in the 1000 Genomes Project database (1000G), the Exome Aggregation Consortium (ExAC), or the Genome Aggregation database (gnomAD). Then, functional impact of remaining variants was assessed using dbNSFP database[7], which compiles multiple prediction tools, including the commonly used SIFT and PolyPhen2 score, to assist the identification of preliminary candidate variants. The DAVID database (https://david.ncifcrf.gov) [8] was used for functional annotation and identification of clinically relevant causative genes for potential pathogenic and modifier variants. The candidate pathogenic variants were further experimentally confirmed by Sanger sequencing (LRP6 forward primer: 5’ATCCCTGGTCTCTGCCTGCTA3’, reverse primer: 5’CGTGGCAGTTGAAATGGTCCT3’).

**Structural modeling**

The crystal structure of the third and fourth β-propeller-EGF-like domain in LRP6: LRP6-PE3PE4 (PDB 4A0P) was used as a template to evaluate the conformation and interaction of the targeted wildtype residue within the protein. Structural modeling of the mutant residue was carried out by in-silico mutagenesis in the pyMol software (The PyMOL Molecular Graphics System, Schrödinger, LLC). Visualization of the tertiary structure of wild-type and mutant LRP6 protein was performed using the Mol* Viewer[9]. The crystal structures of LRP6 β-propeller domains (LRP6-PE1PE2, PDB 3S94; LRP6-PE3PE4, PDB 4A0P) were visualized using Mol* Viewer to examine the structural relevance of reported LRP6 pathogenic variants to its neighboring YWTD motif. In addition, AlphaFold-predicted protein structures of WNT10A (Q9GZT5) and EVC2 (Q86UK5) were also used for assessing the local structural impact of candidate mutations.

**LRP6 plasmids**

To generate a wild-type *LRP6* plasmid, the full-length human *LRP6* coding sequence (NM_002336) was synthesized by ligation (Tsingke Biotech, Beijing, China), and cloned into pcDNA3.1(+) mammalian expression vector using 5’-KpnI and 3’-NotI sites. The LRP6-C1032F and LRP6-G1375D mutant vectors were generated using QuickChange site-directed mutagenesis kit and validated by Sanger sequencing (service provided by Tsingke Biotech).

**Western blot analysis**

Human embryonic kidney cell line 293T cells was maintained in Dulbecco's modified Eagle's medium (DMEM; 4.5g/liter D-glucose) containing 10% fetal bovine serum (Life Technologies, Inc.), 100 units/ml penicillin, and 100μg/ml streptomycin at 37°C and 5% CO2. For transfection, 5×10^5^ 293T cells seeded in 60mm plates were transfected with 4μg of the LRP6-WT, LRP6-C1032F, and G1375D constructs using Lipofectamine 3000 (Invitrogen), respectively. Western blot analysis of LRP6 expression and phosphorylation was performed 48hrs post transfection with Rabbit anti-LRP6 antibody (1:1000, #2560, Cell Signaling) and Rabbit anti-Phospho-LRP6 (Ser-1490) (1:1000, #2568, Cell Signaling) antibody. Equal loading of the samples was confirmed by probing the western blot with Mouse anti-GAPDH (1:10000, #60004-1-Ig, Proteintech) antibody. The band intensity of western blot was further measured with ImageJ software (National Institutes of Health, MD) and the statistical difference was determined by two-tailed unpaired t-test using GraphPad Prism software (San Diego, CA, USA). Statistical significance was set at P < 0.05.

**Data availability**

The LRP6 C1032F mutation identified in this study was submitted to the ClinVar database with accession number SCV003922030. The WES data was submitted to SRA database with accession number PRJNA825233.

**References**

1. Bailey, T.L., et al., *The MEME Suite.* Nucleic Acids Res, 2015. **43**(W1): p. W39-49.

2. Jumper, J., et al., *Highly accurate protein structure prediction with AlphaFold.* Nature, 2021. **596**(7873): p. 583-589.

3. Russ, W.P. and D.M. Engelman, *The GxxxG motif: a framework for transmembrane helix-helix association.* J Mol Biol, 2000. **296**(3): p. 911-9.

4. Matoba, K., et al., *Conformational Freedom of the LRP6 Ectodomain Is Regulated by N-glycosylation and the Binding of the Wnt Antagonist Dkk1.* Cell Rep, 2017. **18**(1): p. 32-40.

5. Yu, M., et al., *Lrp6 Dynamic Expression in Tooth Development and Mutations in Oligodontia.* J Dent Res, 2021. **100**(4): p. 415-422.

6. Zhang, L., et al., *Rare phenotype: Hand preaxial polydactyly associated with LRP6-related tooth agenesis in humans.* NPJ Genom Med, 2021. **6**(1): p. 93.

7. Liu, X., et al., *dbNSFP v4: a comprehensive database of transcript-specific functional predictions and annotations for human nonsynonymous and splice-site SNVs.* Genome Med, 2020. **12**(1): p. 103.

8. Huang da, W., B.T. Sherman, and R.A. Lempicki, *Systematic and integrative analysis of large gene lists using DAVID bioinformatics resources.* Nat Protoc, 2009. **4**(1): p. 44-57.

9. Sehnal, D., et al., *Mol* Viewer: modern web app for 3D visualization and analysis of large biomolecular structures.* Nucleic Acids Res, 2021. **49**(W1): p. W431-w437.
